# Intensive smoking cessation intervention in a nature-based setting: a feasibility study of the Danish gold standard program

**DOI:** 10.3389/fpubh.2024.1470820

**Published:** 2025-01-23

**Authors:** Mie Sylow Liljendahl, Sanne Staal, Hanne Tønnesen

**Affiliations:** ^1^Clinical Health Promotion Centre, WHO-CC, Parker Institute, Bispebjerg and Frederiksberg Hospital, University of Copenhagen, Copenhagen, Denmark; ^2^Center for Health Promotion, Holstebro Kommune, Holstebro, Denmark

**Keywords:** feasibility studies, gold standard program, intensive smoking cessation intervention, national database, nature-based intervention

## Abstract

The implementation of nature-based interventions has demonstrated a positive impact on health outcomes and overall wellbeing. The knowledge of nature-based smoking cessation interventions is sparse but might offer potential benefits for smokers undertaking an intensive smoking cessation program. This study assessed the feasibility in real life of the 6-week Danish Intensive Gold Standard Program (GSP) for smoking cessation in nature compared to the standard setting in primary healthcare. This feasibility study followed up with 81 out of 90 (90%) participants in a nature-based setting and 56,480 out of 58,772 (96%) participants in a standard setting. All participants received the intensive GSP and were registered after providing informed consent in the national Danish STOPbase between 2018 and 2023. Feasibility was assessed using the following indicators: primarily successful quitting after 6 months, secondarily at the end of the 6-week program, as well as compliance and satisfaction—all obtained through interviews. All indicators were similarly high in both settings. After 6 months, 43% had successfully quit in the nature-based setting and 37% in the standardized setting without statistical significance (RR 1.25, 95% CI: 0.80–1.94). The nature-based setting was feasible and appeared to produce similar outcomes as the standard setting for the 6-week intensive GSP in Denmark in real life.

## 1 Introduction

The emergence and popularity of nature-based interventions have increased in recent years, and the implementation of nature-based interventions has demonstrated positive psychological and physiological impacts on individuals with long-term conditions (1). Interventions in nature have, among others, indicated a beneficial effect on cardiovascular diseases (CVDs) and cancer-related outcomes (2) as well as a positive impact on cognitive function and mental health (1, 3). Furthermore, exposure to pictures of nature has been shown to help reduce smoking (4), but the potential for integrating nature-based elements into smoking cessation interventions remains underexplored.

In Denmark, the 6-week intensive Gold Standard Program (GSP) was developed in 1995 and has been implemented nationwide. The GSP is routinely used in primary healthcare and constitutes almost all intensive smoking cessation interventions in Denmark. At the national level, the quality and effect of smoking cessation interventions are registered and followed up in the Danish STOPbase for tobacco and nicotine (5). The effect of the GSP is high in randomized trials and real life; thus, approximately half of the participants have successfully quit smoking by the end of the program, and one of the three is a continuous quitter at 6-month follow-up (6–9). The manual-based program is offered in groups or individually, free of charge, and integrates motivational counseling, patient education, and free pharmaceutical support delivered in 5–6 weekly sessions by certified therapists at approximately 100 STOP-Units distributed nationally (6, 7).

Smoking prevalence in Denmark has decreased from 2020 to 2022 but is still high. In 2022, 13% of the adult population smoked daily—with a higher prevalence among men (15%) than among women (11%). This corresponds to approximately 600,000 daily smokers aged 15 years and older (10). Annually, only 1–2% of them receive intensive smoking cessation interventions (10), which is lower than the 5% recommended in national and international guidelines (5). Despite wishing to quit smoking, many individuals do not participate in cessation programs. Several barriers have been identified, such as a lack of novelty or an unwillingness to tie cessation efforts to medical care (11). Due to the pandemic, several health promotion activities were moved outdoors (into nature) (12). Therefore, the possibility of using nature for smoking cessation was explored and was welcomed by smokers. This emphasizes the need for innovative approaches such as nature-based interventions.

Therefore, the objective of this study was to examine the feasibility of integrating the intensive GSP into a nature-based setting, measured by successful quitting, compliance, and satisfaction, and comparing these outcomes to the standard setting, both within primary healthcare.

## 2 Materials and methods

**Figure 1:**
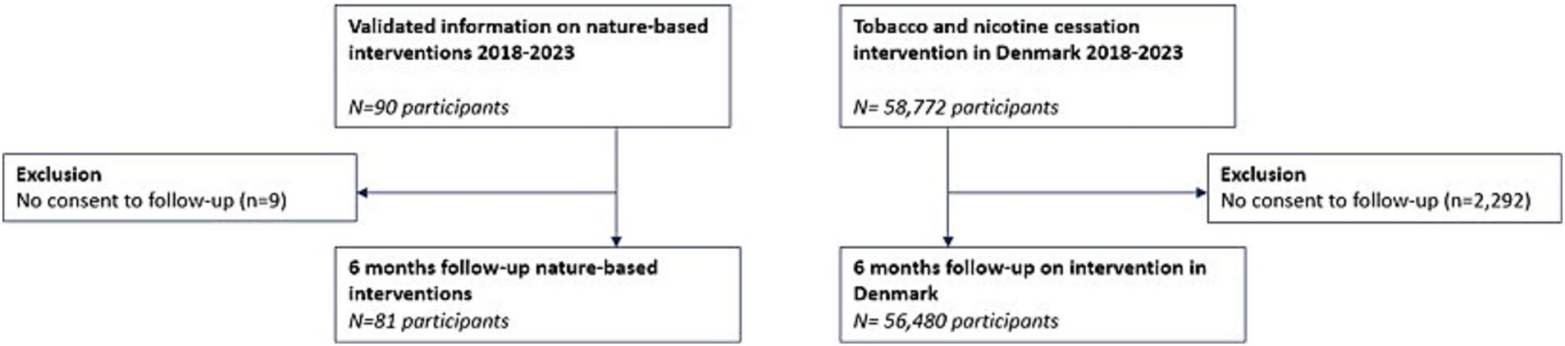
Flowchart of participants’ inclusion in the study.

Overall, 90 smokers chose to participate in the nature-based intensive 6-week GSP instead of the standard setting in Holstebro municipality between 2018 and 2023. All participants gave informed consent for registration in the STOPbase (5, 6) and were followed up at the end of the program. In addition, 81 (90%) consented to the 6-month follow-up. For comparison, all other smokers participating in the GSP at the standard setting in the same period were examined; among 58,772 participants, 56,480 (96%) consented to the 6-month follow-up (Figure 1).

Baseline characteristics revealed that participants in the nature-based setting had a higher use of nicotine replacement therapy. This group included more men, more heavy smokers (measured by pack-years, Fagerström score (13), and cigarettes per day), a higher proportion of participants with shorter education, and a higher unemployment rate compared to the standard GSP setting (Table 1).

**Table 1:** Baseline characteristics of participants in the gold standard program (GSP) delivered in the nature-based setting and the standard setting in Denmark.

	Nature-based GSP *n* (%)	Standard setting GSP *n* (%)
Total participants	90	58,682
Age, median (IQR)	51 (40–62)	54 (42–64)
Male subjects	51 (56.7)	27,476 (46.8)
Smoking profile		
Heavy smoker^1^	79 (87.8)	45,672 (77.8)
≥20 pack-years	64 (71.1)	40,058 (69.3)
Fagerström 7–10 points	37 (41.6)	18,238 (31.9)
≥20 cigarettes per day	22 (36.7)	12,807 (33.3)
Previous attempts to quit	54 (60.0)	34,276 (58.4)
Nicotine replacement therapy	78 (86.7)	35,739 (60.9)
Encouraged by healthcare staff	49 (81.7)	39,805 (95.8)
Living with a smoker	29 (33.0)	16,588 (28.7)
Living alone	25 (28.1)	23,700 (41.0)
Low education level	35 (42.7)	17,813 (32.5)
Unemployment	48 (62.3)	23,276 (41.4)

^1Heavy smoker: At least one of the following: pack-years ≥ 20, Fagerström scores > 6, and/or ≥ 20 cigarettes per day.^

The feasibility was described by several process and effect indicators. The main indicator was continuous quitting after 6 months. The other indicators were compliance measured as meeting adherence, successful quitting at the end of the GSP, and satisfaction.

The study has been reported according to the RECORD guidelines (14), and ChatGPT (GPT-4)^®^ has been used for grammar control.

### 2.1 The nature-based GSP in Holstebro

**Figure 2:**
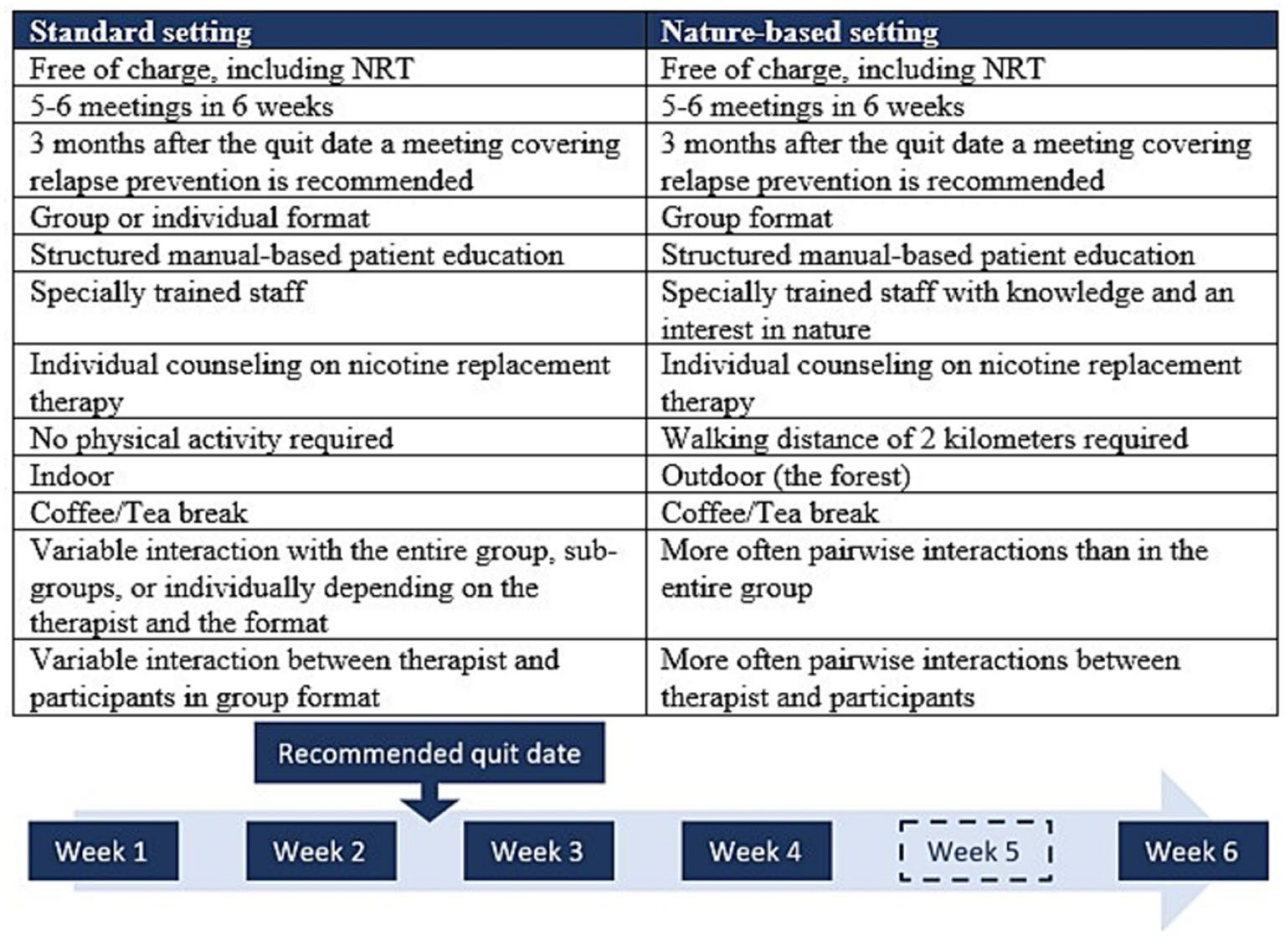
Description of the Gold Standard Program (GSP) delivered in the nature-based setting and the standard setting in Denmark. NRT, nicotine replacement therapy.

Offering the GSP in a nature-based setting involved presenting this alternative format to participants, who could then freely choose between the two settings. The therapists developed an outreach strategy to communicate with potential participants. The concept and target participants of the GSP were similar in the nature-based and the standard settings; however, the nature setting required the ability to walk approximately 2 km, thereby excluding individuals with limited walking ability, e.g., severe cardiac or respiratory diseases (Figure 2). The extra competencies for the therapist included knowledge and interest in nature, such as knowing places to find shelter for exercise on days with bad weather.

The nature-based GSP offers benefits such as additional physical activity, engagement with nature, and fresh air, but also exposes participants to varying weather conditions, including rain, snow, and sunshine. Despite being in a group format, the participants often walked and talked two and two.

Before the session, the group was informed of the meeting point, often located just outside an open forest with suitable walking terrain or other outdoor areas. The GSP and walking started immediately, with the therapist introducing confidentiality and ensuring an open and safe dialogue. Laminated question cards for participants to use in pairs were handed out to initiate and promote an equal dialogue; thus, learning occurred both from therapist to participant and between participants during the walk.

Walking and talking in pairs may create confidentiality that further fosters the sharing of challenges and benefits during the process of change. Nature is symbolically used to address participants’ challenges and problems. As an example, the therapist actively used the nature setting to be aware of the improved sense of smell and other sensory experiences. The nature setting also allowed for a coffee and tea break at a relevant spot in the forest, which might be inside a shelter or cabin in case of rainy weather.

The education elements of the GSP (7) were introduced during the break. The therapist carried the relevant material in a rucksack. In the case of nicotine replacement therapy, the pros and cons were taught, and the products were shown after individually assessing smoking history and nicotine dependence at the first meeting (10), which included time for pairwise discussion. At the second meeting, all the products were shown, and each participant decided if and what they were interested in using. Afterward, the therapist coordinated the delivery with the local pharmacy, as free nicotine replacement therapy is part of the GSP—independent of the setting.

The therapists in the nature setting localized in Holstebro also run the GSP in the standard setting. They have experienced that the nature-based setting works very well. It has added unique nature experiences and promoted self-determination, reflection, and open discussions related to the process of change. The nature setting has been implemented in three other municipalities.

### 2.2 Feasibility measurements

In the STOPbase, smoking was defined as daily combustible smoking of cigarettes, cigarillos, cigars, and/or pipes. The main indicator of the study was the successful quitting of smoking at a 6-month follow-up after the GSP. Successful quitting was defined as complete abstinence from smoking at the end of the 6-week intervention and continuous abstinence at 6-month follow-up. Information on participants’ smoking status was obtained by the Quitline or other trained staff through structured interviews. Participants were contacted up to 4 times between 5 and 7 months after the planned quit date (or at the end of the intervention in cases where no quit date was planned).

The other indicators were compliance, measured as meeting adherence and considered complete at participation in at least 75% of the planned 5–6 meetings, and successful quitting at the end of the GSP. Furthermore, satisfaction was measured on a Likert scale from 1 to 5 and considered high when answering 4 (high) and 5 (very high satisfaction).

All data were obtained by interviewers trained in smoking cessation interventions and registered in the STOPbase.

### 2.3 Statistics

The characteristics and indicators of the two groups were presented as categorical data by numbers and percentages or as continuous data by median and interquartile range (IQR).

The indicators were presented as intention-to-treat and for the completers of the GSP as per-protocol analysis. In addition, the crude relative risk (RR) of the main indicator, successful quitting after 6 months, was compared between the groups and presented with a 95% confidence interval (CI). It was considered significant if the CI did not include the value 1. The sample size in the nature setting was too small for detailed analyses.

Missing data were handled using multiple imputations with chained equations (15). Plausible values for each missing variable were imputed based on a logistic regression model, assuming that the missing values could be accurately estimated from the observed data, in accordance with the missing-at-random mechanism assumption. All data processing and analyses were performed using R statistical software® version 4.3.0 (21 April 2023).

### 2.4 Ethics statement

The Danish STOPbase for tobacco and nicotine was approved by the Danish Data Protection Agency (P-2021-900) and reviewed by the Scientific Ethical Committee of the Capital Region (685 27), with no further comments. Since its inception, the STOPbase has received continuous approval, approximately 10 years at a time, with the most recent approval in 2021.

## 3 Results

The continuous successful quit rates at 6-month follow-up after the GSP did not appear different between the nature and the standard setting, neither as intention-to-treat (43 and 37%) nor as per-protocol analysis (46 and 44%). The crude RR was 1.25 (95% CI 0.80–1.94) (Table 2).

**Table 2:** Successful quit rates and relative risk (RR) for continuous successful quitting at 6-month follow-up after the gold standard program (GSP) delivered in the nature-based setting and standard setting in Denmark.

	*n* (%)	95% CI	RR	95% CI	*p*-value
Nature-based GSP	34 (42.5)	31.8–52.9	1	–	–
Standard setting	21,020 (37.2)	36.8–37.6	1.25	0.80–1.94	0.330

**Figure 3:**
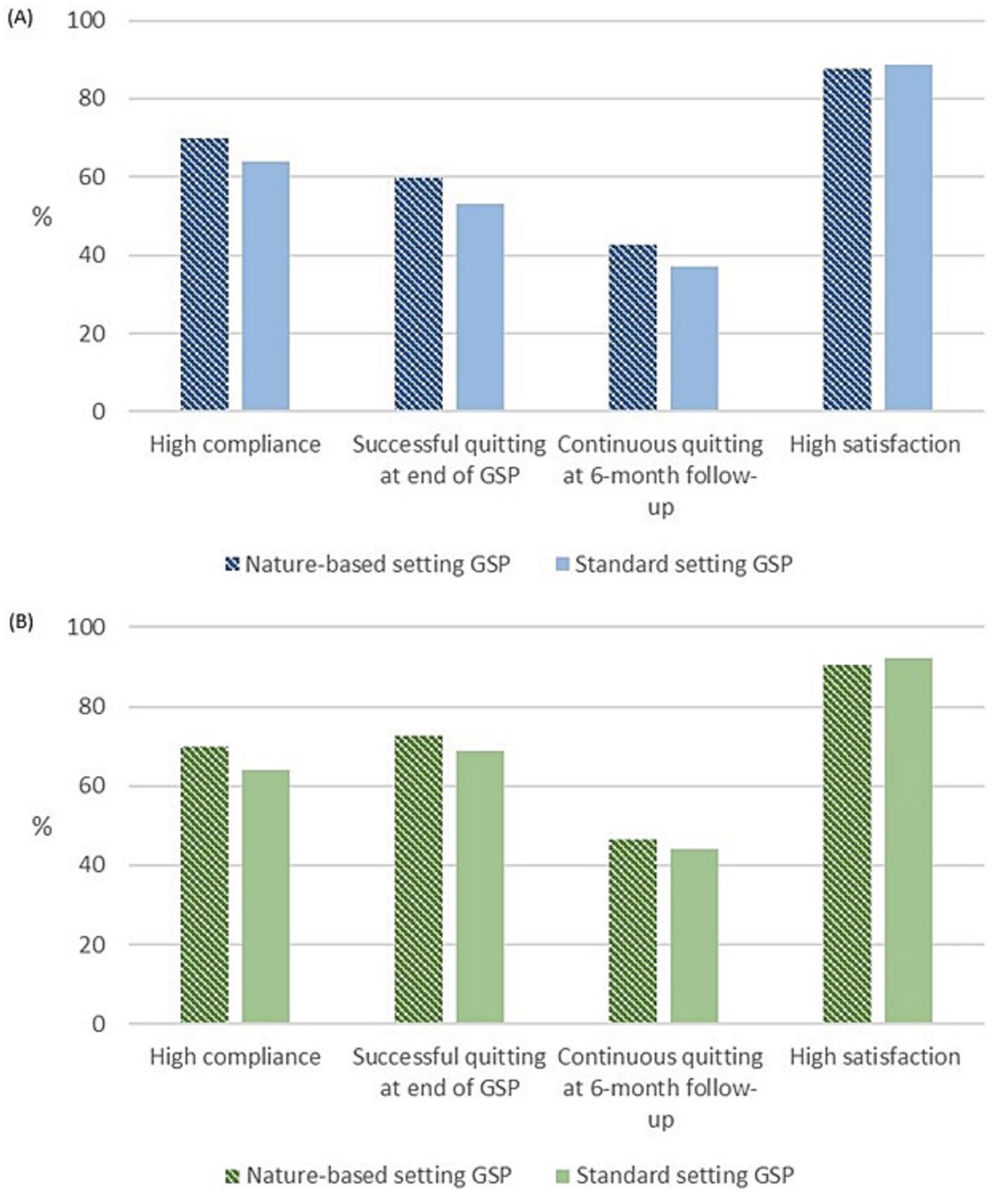
Indicators for the Gold Standard Program (GSP) delivered in the nature-based setting and the standard setting in Denmark; **(A)** all participants as intention-to-treat and **(B)** participants complied ≥75% meeting adherence of the intervention (per protocol).

The other indicators also appeared to reach similar levels across the groups in intention-to-treat (Figure 3A) and per-protocol (Figure 3B) analyses.

## 4 Discussion

This study tested the feasibility of an evidence-based intensive smoking cessation intervention in a different setting (16) and showed that the nature-based setting was feasible for the delivery of the intensive 6-week GSP. The successful quit rates at the end of the program and at the 6-month follow-up, along with compliance and satisfaction, were similar to the strong results obtained in the standard setting. Acceptability of the changed intervention setting was observed among both participants and therapists.

The feasibility of a nature-based smoking cessation intervention, such as the GSP, has not previously been tested; however, the literature shows positive experiences with other health-promoting treatments or interventions for other risk factors in nature-based settings (1, 2). In addition, nature-based interventions have shown potential for the treatment and prevention of mental health issues (3, 17, 21). A recent study reported a positive impact of nature-based education on health-related quality of life in low-income youth (18), suggesting that the unused potential of such approaches could be significant. Furthermore, an outdoor format can be particularly useful during pandemics such as COVID-19, as it allows for safer, socially distanced interactions. Several other studies have evaluated nature-based interventions (1–3, 17–19). However, the designs, the focus, and the target groups differ from our study. Nevertheless, they all suggest nature-based intervention as a relevant setting for intervention on health behaviors.

However, the practical aspects of delivering the nature-based GSP are different from the standard setting. In this study, the therapists had to bring all necessary equipment and supplies for the participants and consider weather conditions. Therapist them-selves needed to be able to walk and be familiar with the local nature and its history. They also had to be open to integrating the natural surroundings and the related flexibility into the GSP delivery. Pairwise discussions might require more interaction between therapists if pairs are not functioning optimally. Additionally, participants should be able to walk a few kilometers, and safe forest terrain should be accessible. The interest in walking a few kilometers at each meeting may indicate a generally healthier population compared to the overall GSP population, potentially contributing to better cessation rates. The smoking profile of the participants in the nature-based setting showed more heavy smokers but high compliance and use of nicotine replacement therapy, which are known predictors for successful cessation.

Based on the experience from Holstebro, the nature setting is now offered in several other municipalities in Denmark, and the potential for further implementation is increased. Safe outdoor terrain is relatively easy to find in a small country such as Denmark, with well-regulated safe forests, numerous open natural areas, short distances, hills instead of high mountains, and very few dangerous wild animals. Therefore, adapting this approach to other countries would require modifications to accommodate local challenges, while still maintaining the core components of the nature-based setting for intensive smoking cessation intervention. Thus, the study has several limitations for generalization, and further studies of nature-based settings should be conducted in different geographical contexts.

The study has strengths as well. Among others were the unchanged and high-quality data collection via the national STOPbase, the well-defined and characterized GSP, and the study population as a whole. The consent to 6-month follow-up was very high. Through the interview of one of the therapists, the nature setting has been explained in detail, ensuring the certainty of the extent of following the concept of the GSP.

Several biases must also be considered, as only interested individuals participated in the nature setting and may therefore be highly motivated in this setting. The therapists often both administered the intervention and collected the baseline data. However, the follow-up at 6 months is mostly performed by trained therapists from Quitline or others. Furthermore, collecting data using interviews is more reliable compared to participants filling in questionnaires separately by themselves (20). The small sample size in the nature-based setting leads to an inability to adjust for potential confounders, and the sample only represented just one of the 98 municipalities in Denmark, introducing selection bias. The lack of generalization and small sample size limit the applicability of the results to other populations or settings. Feasibility studies are often designed to test an intervention on a smaller scale, with limited statistical power, to assess whether it can be delivered effectively in a given setting compared to usual practice. Therefore, the effect of nature-based interventions for smoking cessation needs to be tested in sizeable studies of high-quality designs such as cohorts or randomized trials, preferably with nested interviews. Preferences for the structure of the nature-based setting may have influenced both the participants’ engagement and the effect. Although no significant differences were found in successful cessation rates, these findings should be interpreted cautiously. The small sample size, potential selection bias, and inability to adjust for potential confounders in addition to factors such as the higher use of nicotine replacement therapy and greater compliance in the nature-based setting may partly explain the more favorable outcomes observed. This study does not solve the general challenges for participation, which should be elucidated further in relation to introducing nature-based interventions in general.

In conclusion, the nature-based setting appeared to be as feasible as the standard setting for the intensive GSP. It may serve as an attractive supplement to the clinical setting for interested groups with the ability to walk a few kilometers.

## Data Availability

The anonymized data and statistical codes are available from the corresponding author upon reasonable request mie.sylow.liljendahl.01@regionh.dk.
